# Genome-wide selection analysis identifies genes associated with mandibular defects in Duolang sheep

**DOI:** 10.3389/fvets.2025.1714754

**Published:** 2026-01-05

**Authors:** Hang Cao, Qiuming Chen, Ling-Ling Liu, Wu-Jun Liu

**Affiliations:** College of Animal Sciences, Xinjiang Agricultural University, Ürümqi, Xinjiang, China

**Keywords:** candidate genes, Duolang sheep, FST, mandibular prognathism, nucleotide diversity (*π*)

## Abstract

**Introduction:**

Brachycephaly is characterized by a conspicuously shortened craniofacial structure. While it is a defining trait in certain species such as Bulldogs, Persian cats, Niata cattle, Anglo-Nubian goats, and Middle White pigs, it may also represent a pathological condition. In Duolang sheep, brachycephaly is strikingly pronounced and may arise from distinct genetic and developmental mechanisms linked to breed history. This condition, often accompanied by mandibular prognathism, predisposes affected animals to health problems including brachycephalic airway obstruction syndrome, nasal blockage with dyspnea, and feeding difficulties. Duolang sheep, a dominant indigenous breed in southern Xinjiang, China, comprise approximately two million individuals, nearly 10% of which exhibit this heritable defect. Such a high prevalence poses a significant challenge to genetic improvement programs in local populations. Elucidating the genetic basis of this phenotype and identifying candidate genes is therefore essential.

**Methods:**

To characterize the craniofacial phenotype, we obtained X-ray measurements of maxillary length, mandibular length, and incisor angle. Affected sheep displayed significantly shorter maxillae and longer mandibles compared with controls (*p* < 0.05), whereas incisor angle showed no significant difference. Here, we performed whole-genome resequencing of two phenotypically distinct Duolang sheep cohorts and analyzed genomic architecture and variation patterns using a selective sweep approach.

**Results:**

Candidate regions under strong selection were identified by Fst and *π*-ratio analyses. Gene annotation revealed functionally relevant candidates associated with skeletal growth and development (MTX2, FGF12, IGF, PPARGC1A, LIPC, LY86, and IL33).

**Discussion:**

These findings provide new insights into the genetic basis of brachycephaly in Duolang sheep and offer strategies for genetic improvement, contributing to broader research on skeletal development and growth regulation in livestock.

## 1 Introduction

Animal domestication is often accompanied by morphological changes, notably shortening of the snout relative to the cranium, a phenomenon linked to domestication syndrome (1–5). This has been hypothesized to result from neural crest–driven effects associated with selection for tameness (6), although this remains debated (7). Such cranial modifications differ from the extreme snout shortening and facial tilting observed in specific breeds, such as bulldogs, pugs, and Persian cats, collectively referred to as brachycephalic (8, 9). In some species, such as bats, brachycephaly may confer adaptive advantages by enhancing the functional efficiency of oronasal structures (10). In domesticated animals, however, extreme brachycephaly, including mandibular prognathism, is often associated with functional impairments or pathological conditions such as airway obstruction, respiratory distress, and feeding difficulties (11, 12).

**Figure 1:**
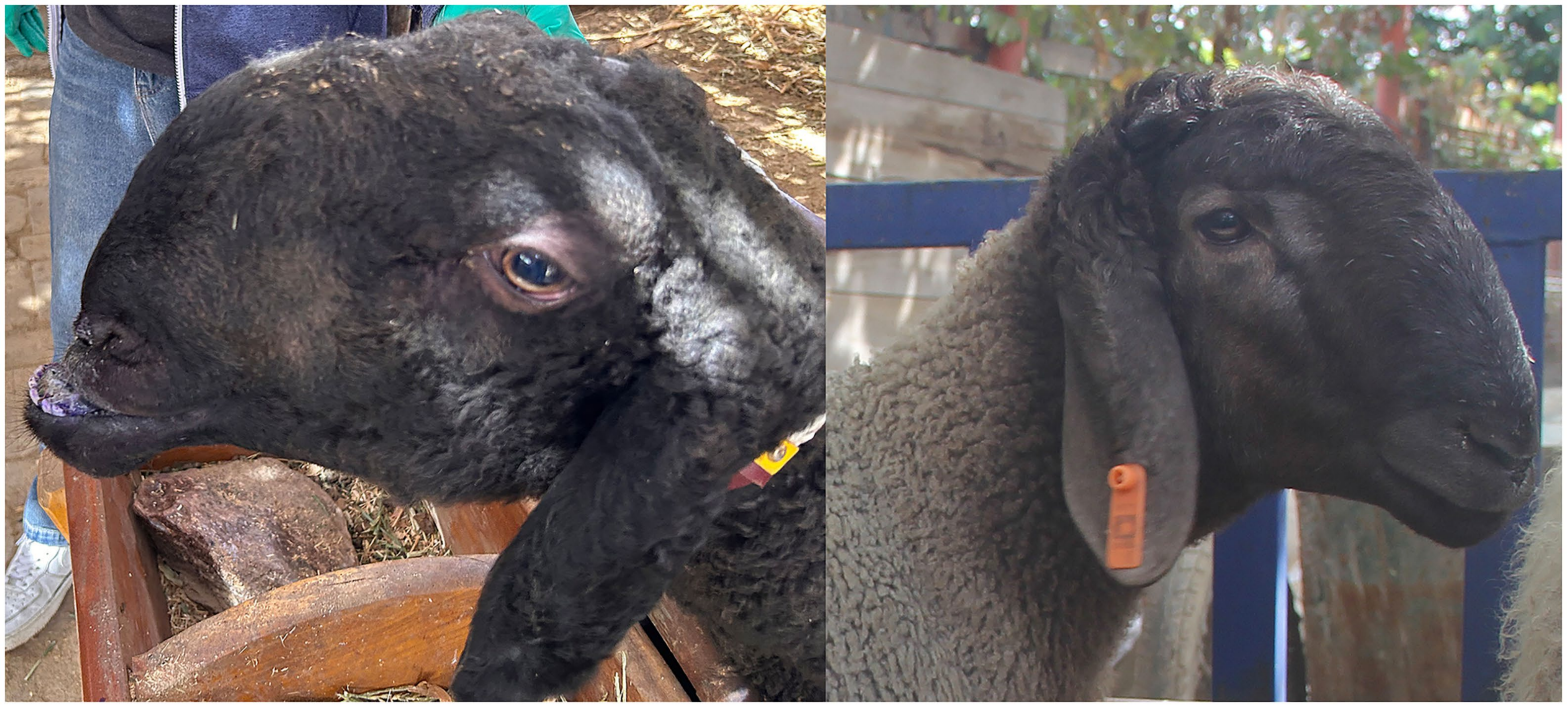
Mandibular prognathism sheep and normal sheep.

Duolang sheep are a predominant indigenous breed in Xinjiang, China, with a population of approximately two million. Adapted to the arid climate of southern Xinjiang, they play a critical role in regional meat production and local livelihoods. Genetic defects such as mandibular prognathism significantly impair production traits, including reproduction and meat yield, necessitating genomic interventions to eliminate this defect. While brachycephaly has been extensively studied in other domesticated species, it has not been reported in sheep. In Duolang sheep, mandibular prognathism is pronounced and likely arises from unique genetic and developmental mechanisms tied to breed history (Figure 1).

Genomic technologies have revolutionized livestock improvement by enabling the identification and elimination of deleterious variants associated with genetic defects. To this end, selective sweep analyses—leveraging metrics such as the fixation index (FST) and nucleotide diversity (*π*)—offer robust methods for detecting selection signatures that pinpoint genomic regions harboring such variants (13). For example, selective sweep analyses in combination with whole-genome sequencing of diverse dog breeds identified regions of reduced heterozygosity associated with brachycephaly, including a missense mutation in BMP3 on chromosome 32 that contributes to skull shortening along the brachycephalic-dolichocephalic continuum in breeds such as Bulldogs and Pekingese (14). Similarly, whole-genome sequencing and quantitative trait locus mapping in pedigree and mixed-breed dogs pinpointed a retrotransposon insertion in an intron of SMOC2 on chromosome 1, which disrupts splicing and reduces gene expression to promote facial retrusion and snout shortening in brachycephalic breeds including Pugs and Boston Terriers (15).

## 2 Materials and methods

### 2.1 Sample

**Figure 2:**
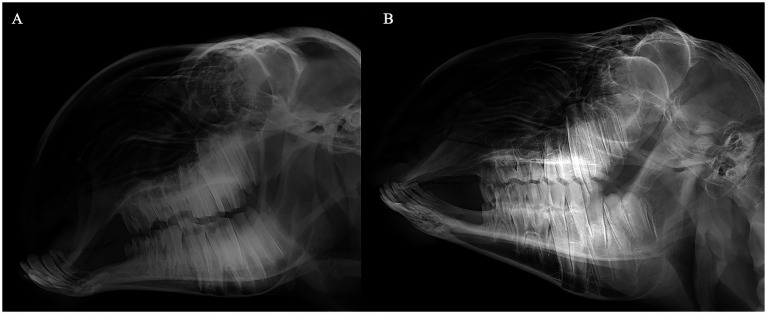
Comparative cephalometric radiographs of Duolang sheep. **(A)** Affected sheep with mandibular prognathism. **(B)** Phenotypically normal healthy sheep.

Blood samples were collected from 175 Duolang sheep at the Stock Breeding Farm in Maigaiti County, Kashgar Prefecture, Xinjiang Uyghur Autonomous Region, China. The cohort comprised 145 individuals with mandibular prognathism and 30 phenotypically normal individuals. All subjects underwent radiographic examination using X-ray imaging systems to capture cranial structures, followed by quantitative measurement of phenotypic parameters based on the obtained images (Figure 2). The X-ray images of the sheep were taken using a medical X-ray collimator (standard number: YZB0671-2007). Imaging Protocol and Calibration: Lateral cranial X-rays were taken for all animals using the same system (YZB0671-2007) with standardized positioning (Frankfort plane horizontal, teeth in natural occlusion, minimal head rotation). A 10 cm radiopaque ruler was included for scale calibration. Images with motion blur or head rotation over 5° were discarded and retaken when possible. Pixel-to-millimeter conversion was based on the in-image ruler. Landmark Definitions and Measurements: Maxillary Length (ML) is measured from the midpoint of the anterior nasal bone to the junction of the posterior maxilla and zygomatic bone. Mandibular Length (JL) is the distance from the midpoint of the mandibular front cutting groove to the posterior edge of the mandibular angle. Incisor Angle (IA) is the angle between the occlusal surfaces of the upper and lower incisors. The protocol ensures strict control: heads are fixed in a standard bracket, the scanner-head distance is 50 cm, and ambient light is 300–500 lux. Approximately 2 mL of blood was drawn from the jugular vein into 5-mL EDTA tubes. Samples were transported on ice and stored at −20 °C until processing. All procedures were conducted in accordance with the Regulations for the Administration of Affairs Concerning Experimental Animals of China and were approved by the Animal Care Committee of Xinjiang Agricultural University (Approval No.: 2021103).

### 2.2 DNA extraction and sequencing

Genomic DNA was extracted using a standard phenol-chloroform protocol. DNA quality was evaluated via 1% agarose gel electrophoresis, and concentrations were quantified using a Qubit fluorometer (Invitrogen, Carlsbad, CA, USA). Purified DNA samples were shipped on dry ice to Novogene Bioinformatics Institute (Beijing, China). Following fragmentation, adapter ligation, and PCR amplification, 350-bp libraries were constructed for each individual. Sequencing was performed on an Illumina NovaSeq 6,000 platform (Illumina Inc., San Diego, CA, USA) in 2 × 150-bp paired-end mode.

### 2.3 Read mapping and variant detection

Sequenced reads were processed using Trimmomatic v0.39 (16) with the parameters “LEADING:20 TRAILING:20 SLIDINGWINDOW:4:20 MINLEN:90” to remove low-quality bases. The filtered reads were aligned to the reference genome assembly (ARS-UI_Ramb_v3.0) using the BWA-MEM algorithm v0.7.17-r1188 (17) with default settings. The resulting alignment files were coordinate-sorted by chromosome using Samtools v1.17 (18), duplicates were marked using the MarkDuplicates module in GATK v4.4.0.0 (19), and mate-pair information was synchronized using GATK’s FixMateInformation module.

Variant calling was performed for each individual using GATK’s HaplotypeCaller module, followed by merging variant files with the CombineGVCFs module. Joint genotyping was conducted with the GenotypeGVCFs module. Hard filtering criteria were applied using the VariantFiltration module, with thresholds as follows: for SNPs, “QD < 3.0 || FS > 30.0 || SOR > 4.0 || MQ < 30.0 || MQRankSum < −10.0 || QUAL < 50.0 || ReadPosRankSum < −5.0”; and for InDels, “QD < 3.0 || FS > 100.0 || ReadPosRankSum < −10.0.” Bi-allelic variants were retained using the SelectVariants module by requiring allele count > 0 and allele frequency < 1 to exclude monomorphic and fixed sites. To further improve genotype-level accuracy, additional filtering was applied using an in-house Perl script in conjunction with BCFtools v1.17 (20). Specifically, we removed genotypes with genotype quality (GQ) < 20 and filtered based on read depth (DP), excluding genotypes with depths <1/3 × or >3 × the individual’s mean whole-genome coverage. For heterozygous genotypes, we further required an allele depth ratio (AD) ≥ 0.2 to reduce the likelihood of sequencing artifacts.

### 2.4 Detection of candidate genes

Candidate genes were defined as those located within ±50 kb of SNPs under selection, based on the reference genome annotation.

### 2.5 Detection of selective signatures

Population genetic differentiation was assessed using genome-wide FST and nucleotide diversity ratios (*π*_case/π_control) in a sliding-window analysis (100 kb windows, 50 kb step) (21, 22). Outlier windows within the top 1% of both FST and π-ratio distributions were considered as putative selection signals.

The sheep genome is about 2.6 Gb, and linkage disequilibrium (LD) usually decreases to r^2^ ≈ 0.2 within 50–200 kb, varying by breed. Thus, we employed 100 kb windows with 50 kb steps to integrate signals across LD blocks while preserving resolution, a method often used in sheep selective sweep studies.

Due to the imbalance in the case–control cohorts (145 cases vs. 30 controls), we assessed robustness by: (i) Down-sampling cases to 30 to match controls, recalculating Weir and Cockerham FST and the *π* ratio in 100 kb windows (50 kb step) over 100 iterations, and measuring concordance with Spearman correlation and top 1% window overlap; (ii) Estimating standard errors and 95% CIs for window statistics using a chromosome-wise block-jackknife with 200 kb blocks.

### 2.6 Functional enrichment analysis

Genes overlapping selective regions were considered candidates under positive selection. GO and KEGG enrichment analyses were performed using the ClusterProfiler package (23), with adjusted *p* < 0.05 considered significant. Enriched pathways highlighted the key biological processes and signaling networks associated with candidate genes.

## 3 Results

### 3.1 Descriptive statistics

Normal Duolang sheep exhibited a greater mean maxillary length (156.92 ± 23.04 mm) than brachycephalic sheep (149.12 ± 22.89 mm), whereas brachycephalic sheep showed a longer mandibular length (211.46 ± 23.19 mm) compared with normal individuals (197.43 ± 32.41 mm). The mandibular incisor protrusion angle was comparable between groups (104.43 ± 7.04° vs. 104.66 ± 8.31°). *T*-tests indicated significant differences in maxillary and mandibular lengths (*p* < 0.05), but not in incisor protrusion angle (*p* > 0.05) (Table 1; Figure 2).

**Table 1:** Summary statistics of selection signatures traits within and across breeds.

Sheep cranial measurements (mm)	Average ± SD		Mean difference	*p*
	Case	Control		
Maxillary length (mm)	149.12 ± 22.89	156.92 ± 23.04	−7.80	0.036
Mandible length (mm)	197.43 ± 32.41	211.46 ± 23.19	−14.03	0.046
Angle (°)	104.43 ± 7.04	104.66 ± 8.31	−0.23	0.87

### 3.2 Genome resequencing and genetic variation

Whole-genome resequencing of 145 cases and 30 controls generated approximately 5,250.79 Gb of data, with an average sequencing depth of 10 × per sample, 35,663,299 SNPs and 4,161,247 Indel.

### 3.3 Population genetics analysis

**Figure 3:**
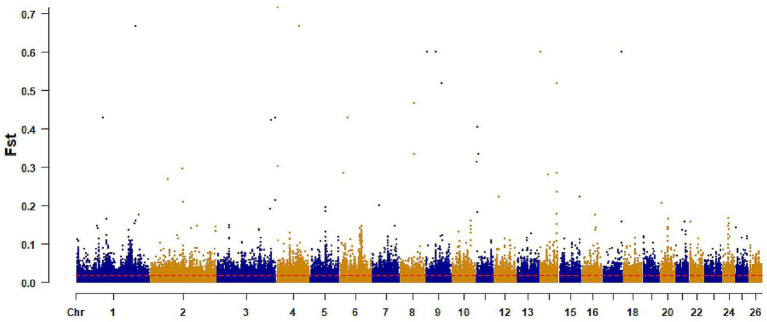
Selection signal of FST. The x-axis: represents chromosomes, the y-axis represents FST values, and the ref. dashed line represents the significance threshold.

**Figure 4:**
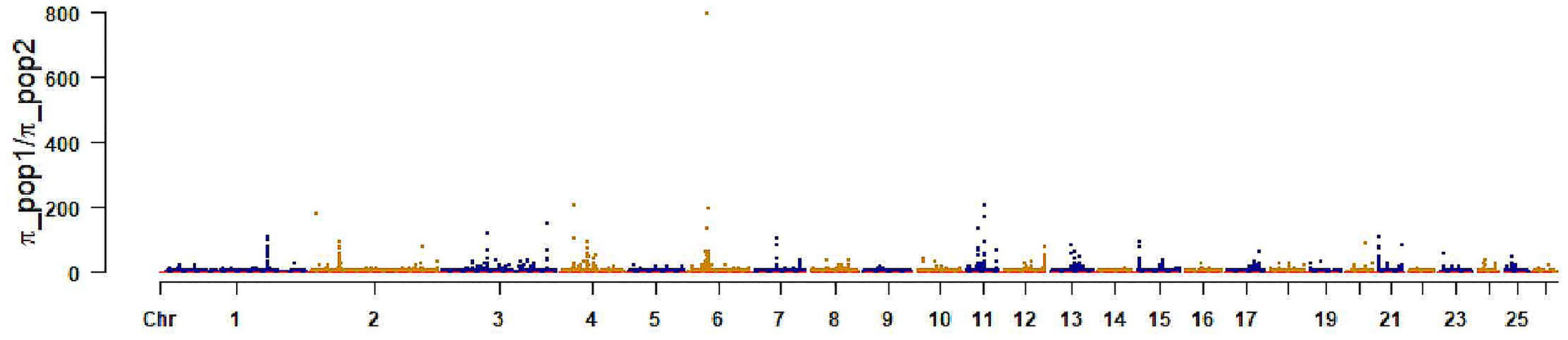
Selection signal of *π* ratio. The x-axis represents chromosomes, the y-axis represents (πcontrol/πcase), and the red dashed line represents the significance threshold.

**Figure 5:**
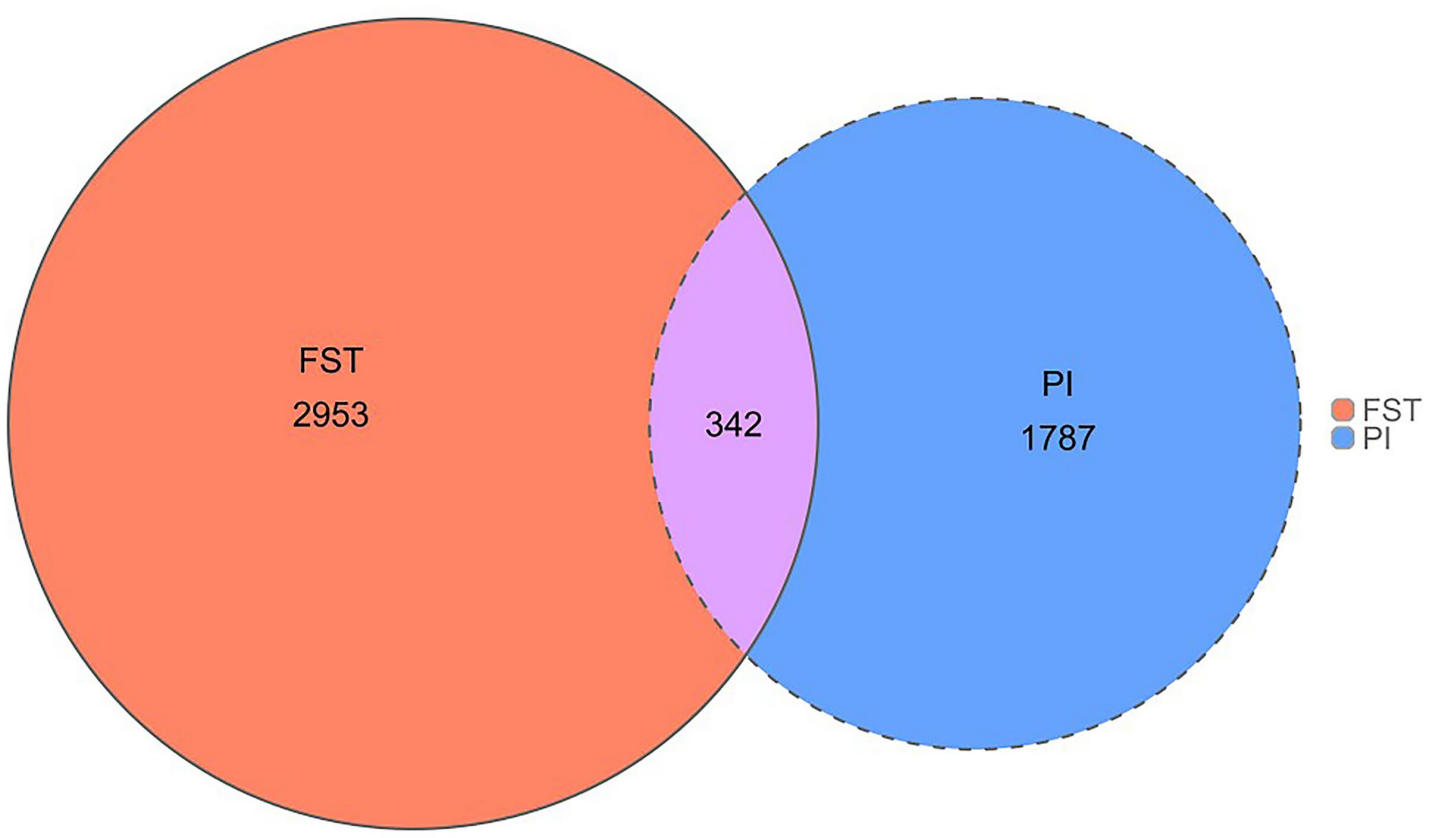
Venn diagram of the two methods.

The healthy and brachycephalic groups were analyzed separately using pairwise FST and *π* ratio statistics in a sliding-window approach across the autosomes to detect selective sweep regions, The top 1% FST windows yielded 5,509 loci, corresponding to 2,953 annotated genes. The top 1% π windows identified 5,503 loci and 1,787 genes (Figures 3, 4). Intersecting the two sets resulted in 342 shared candidate genes (Figure 5).

### 3.4 Gene function enrichment analysis

**Figure 6:**
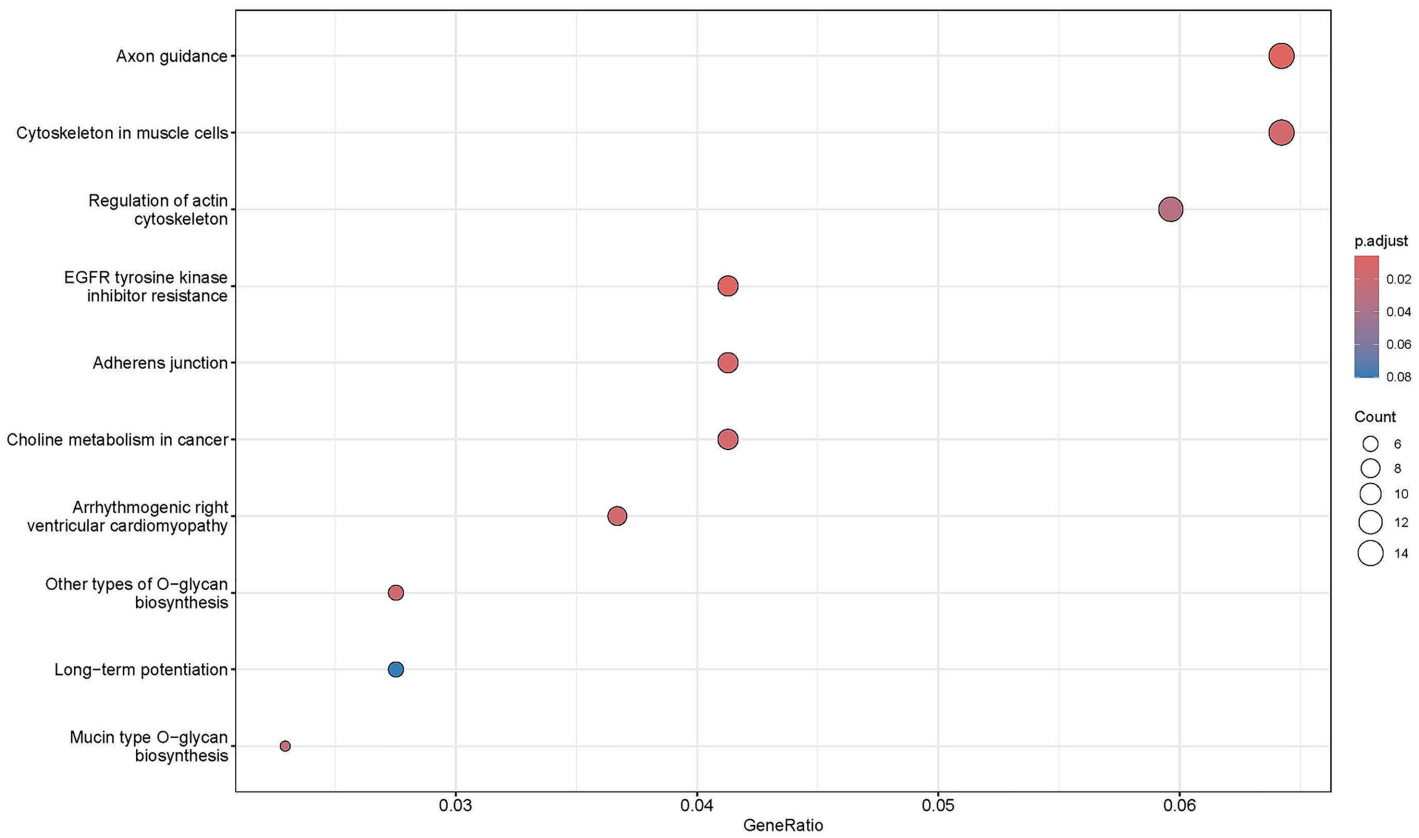
GO enrichment results.

Gene ontology (GO) and Kyoto Encyclopedia of Genes and Genomes (KEGG) enrichment analyses were performed on 342 candidate genes shared between Duolang sheep with two methods. GO analysis revealed significant enrichment in pathways related to neural activity regulation, suggesting a potential role in controlling mandibular prognathism in Duolang sheep. Specifically, enriched GO terms included processes such as negative regulation of neural signaling and microtubule motor activity, as well as cellular components like the actin cytoskeleton (Figure 6).

**Figure 7:**
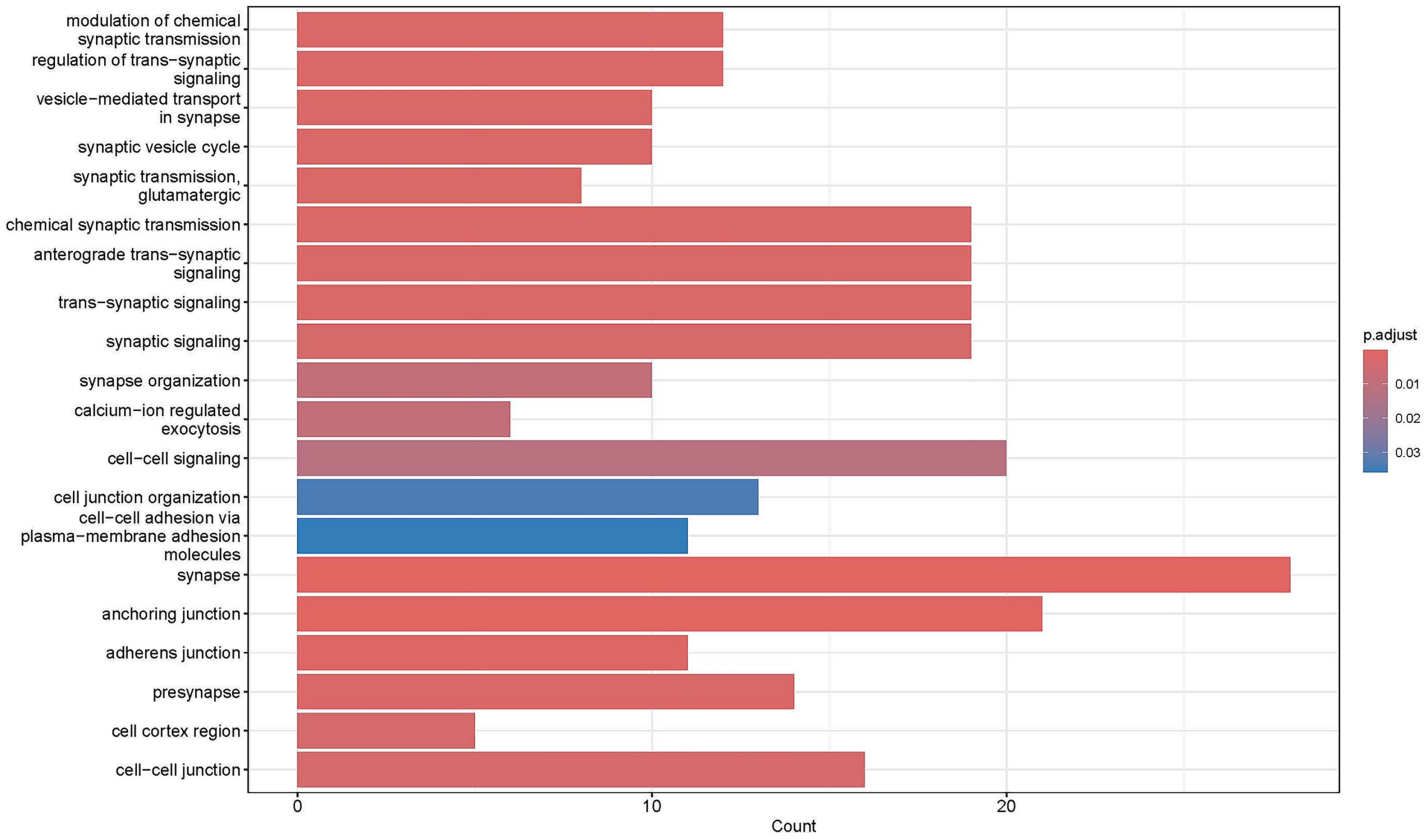
KEGG enrichment results.

KEGG pathway analysis identified 39 significantly enriched pathways, categorized into five functional groups: signal regulation, cancer-related pathways, cellular structure and metabolism, neuro-endocrine regulation, and infection/stress response. Key pathways included those associated with neural conduction regulation, insulin secretion and metabolism, and endometrial hormone signaling. These findings indicate that neural activity and neuro-endocrine regulation may underpin the genetic basis of mandibular prognathism in Duolang sheep, providing insights for targeted genetic improvement (Figure 7).

## 4 Discussion

### 4.1 Craniofacial alterations underlying mandibular prognathism in Duolang sheep

The causes of cranial development defects can be divided into three categories in Duolang sheep with mandibular prognathism. First, prognathism may result from a shortened maxilla (short face) with a normal mandible. Second, it may result from an elongated mandible with a normal maxilla. Third, both the maxilla and mandible are altered, with the maxillary change being more pronounced. Phenotypic data analysis (Figures 1, 2; Table 1) showed that the maxilla of affected Duolang sheep was significantly shorter than that of healthy individuals (*p* < 0.05). The mandible was also shorter in affected sheep compared to healthy ones, indicating that the overall skull size was reduced (*p* < 0.05). Moreover, the reduction in cranial length was more severe than that of the mandible. These results suggest that concurrent shortening of both the maxilla and mandible, with greater reduction in the maxilla, may underlie the occurrence of mandibular prognathism in Duolang sheep.

In dogs, extreme brachycephaly is characterized by a high cranial index (CI), calculated as skull width divided by skull length and expressed as a percentage (24). Selective breeding for extreme brachycephaly has led to a conformation-related respiratory disorder, termed brachycephalic obstructive airway syndrome (BOAS) (25). Affected dogs exhibit upper airway deformation due to skull shortening without proportional reduction in soft tissues, along with additional abnormalities in the lower respiratory tract (26). Unlike brachycephalic dogs, Duolang sheep with prognathism were initially thought to be affected solely by mandibular elongation without maxillary changes, suggesting no predisposition to respiratory difficulties. However, field investigations revealed that prognathic Duolang sheep frequently develop obstructive respiratory syndrome, consistent with our experimental findings. We compared 342 shared candidate genes/regions with Sheep QTLdb annotations for cranial and body traits. Cross-species analysis with dog, cattle, and human studies identified conserved genes like RUNX2, COL1A1, and FOXL2, but typical dog brachycephaly genes (BMP3, SMOC2) were not prominent, suggesting species- and breed-specific differences (24, 25).

The imbalance in sample size (145 cases versus 30 controls) can lead to increased variance and diminished statistical power, particularly when analyzing weak or narrowly defined signals using frequency-based statistics. To address this issue, we employed matched sampling, down-sampled to create balanced subsets, and utilized block-jackknife methods to estimate uncertainty. Our focus was on identifying candidates that demonstrated robustness across these analytical approaches. Nonetheless, we recognize the possibility that some regions with moderate effects may have been overlooked due to the limited number of controls. Future research will prioritize the expansion of a rigorously matched control cohort to address this limitation.

### 4.2 Candidate genes for craniofacial development

Our study identified several candidate genes associated with the brachycephalic phenotype and mandibular prognathism in Duolang sheep, including RUNX2, FGF12, FOXL2, MTX2, COL1A1, and MYO5A. These genes are well-documented in craniofacial and skeletal development across species, reinforcing their relevance to the observed phenotypes. RUNX2, a key regulator of osteoblast differentiation, governs cranial suture formation and bone development. Mutations in RUNX2 cause cleidocranial dysplasia in humans, marked by delayed cranial suture closure, abnormal craniofacial morphology, and mandibular anomalies (27). Similarly, COL1A1, which encodes type I collagen, is critical for bone matrix formation, and its disruption leads to osteogenesis imperfecta, characterized by skull deformities (28). FGF12, a fibroblast growth factor, modulates craniofacial morphogenesis by interacting with FGFR1/2 receptors to regulate maxillary and mandibular prominence fusion and growth (29). In Duolang sheep with mandibular prognathism, altered FGF12 expression may suppress FGF8/FGF10 signaling in the maxilla, reducing maxillary growth, while leaving FGF9/FGF18-mediated mandibular growth intact, thus contributing to relative mandibular elongation (30, 31). FOXL2, a transcriptional regulator, influences cranial suture fusion and facial morphogenesis (32). MYO5A, involved in intracellular trafficking, is linked to craniofacial defects in mouse and human studies (33). ACTN1, an actin-binding protein, contributes to cytoskeletal organization during osteogenesis (34). Additionally, MTX2 contributes to skeletal development, with a splice-site variant (c.543 + 1G > T) causing mandibular hypoplasia in humans, characterized by facial dysmorphism and growth retardation (35). Pathway enrichment analysis identified these genes within growth-related networks, including Wnt, PI3K-Akt, and ErbB signaling, which regulate osteoblast proliferation and inhibit apoptosis, supporting craniofacial development (36–38). Our findings converge on skeletal-development pathways also implicated in dog and human craniofacial variation, while canonical dog brachycephaly loci (BMP3, SMOC2) were not top signals here, underscoring species- and breed-specific mechanisms. For practical deployment, we propose a marker-assisted scheme focusing on high-confidence regions validated across methods and robustness checks. Recommended steps: (i) genotype prioritized SNPs (e.g., missense or promoter-proximal variants near RUNX2, FGF12, MTX2) using a low-cost panel; (ii) compute an exclusion index for breeding animals combining genotype dosage and pedigree inbreeding; (iii) exclude or de-prioritize sires/dams carrying risk alleles while maintaining diversity by rotating unrelated carriers and monitoring effective population size. Our findings converge on skeletal-development pathways also implicated in dog and human craniofacial variation, while canonical dog brachycephaly loci (BMP3, SMOC2) were not top signals here, underscoring species- and breed-specific mechanisms. For practical deployment, we propose a marker-assisted scheme focusing on high-confidence regions validated across methods and robustness checks. Recommended steps: (i) genotype prioritized SNPs (e.g., missense or promoter-proximal variants near RUNX2, FGF12, MTX2) using a low-cost panel; (ii) compute an exclusion index for breeding animals combining genotype dosage and pedigree inbreeding; (iii) exclude or de-prioritize sires/dams carrying risk alleles while maintaining diversity by rotating unrelated carriers and monitoring effective population size. These findings suggest that the brachycephalic phenotype in Duolang sheep shares a partially conserved genetic basis with craniofacial disorders in other species, providing insights into the molecular mechanisms underlying mandibular prognathism.

### 4.3 Candidate genes for neural regulation influencing craniofacial development

We also identified genes involved in neural regulation that may indirectly influence the brachycephalic phenotype in Duolang sheep through neural crest cell-mediated effects, given the pivotal role of neural crest cells in craniofacial morphogenesis. Key candidates include GABRG3 and RORA. GABRG3 encodes a GABA receptor subunit that mediates inhibitory neural signaling, which can modulate neural crest cell migration and differentiation during embryogenesis (39). Altered GABRG3 expression may affect neural crest-derived craniofacial tissues, potentially contributing to mandibular prognathism or brachycephaly. RORA, a nuclear receptor regulating circadian rhythms and cellular differentiation, is expressed in neural crest-derived tissues and has been associated with cranial suture formation and facial morphology in mice (40). In Duolang sheep, RORA may influence craniofacial development by modulating neural crest cell differentiation, particularly under environmental pressures such as the arid Xinjiang climate. Selective sweep analyses showed stronger selection signatures for GABRG3 and RORA in brachycephalic sheep compared to normal individuals, suggesting that artificial selection for shorter heads and arched noses may have amplified these neural-related genes, indirectly shaping craniofacial architecture. Pathway enrichment analysis indicated that GABRG3 and RORA are enriched in Circadian Entrainment and Long-Term Potentiation/Depression (LTP/LTD) pathways, which regulate neural signaling and cellular differentiation critical for neural crest function (41). We detected selection signals near neural regulation genes including GABRG3 and RORA. Given the role of neural crest cells in craniofacial morphogenesis, these findings are intriguing but remain hypothesis-generating. Our data do not establish a direct mechanistic link between these loci and mandibular prognathism. Instead, we propose that variation in neural signaling pathways could indirectly modulate craniofacial development through effects on cell migration or differentiation, a possibility to be tested in future expression and functional studies. Accordingly, we refrain from inferring causality and highlight these as secondary candidates pending additional evidence.

## 5 Conclusion

Mandibular prognathism in Duolang sheep, driven by greater maxillary than mandibular shortening, suggests conserved mechanisms of craniofacial defects across species. Candidate genes directly regulating skeletal development and indirectly influencing morphogenesis via neural crest cells provide novel insights into the molecular basis of mandibular prognathism, informing targeted genomic interventions to enhance the health and productivity of this regionally significant breed.

## Data Availability

The datasets presented in this study can be found in online repositories. The names of the repository/repositories and accession number(s) can be found at: https://www.ncbi.nlm.nih.gov/, PRJNA1177205.
